# Acute Promyelocytic Leukemia Presenting With a Myeloid Sarcoma of the Spine: A Case Report and Literature Review

**DOI:** 10.3389/fonc.2022.851406

**Published:** 2022-03-04

**Authors:** Xuemei Shu, Qiuling Wu, Tao Guo, Hua Yin, Jingdi Liu

**Affiliations:** Institute of Hematology, Union Hospital, Tongji Medical College, Huazhong University of Science and Technology, Wuhan, China

**Keywords:** acute promyelocytic leukemia (APL), myeloid sarcoma (MS), spine, diagnosis, chemotherapy

## Abstract

Myeloid sarcoma is a rare extramedullary tumor of immature myeloid cells. Certain known acute myeloid leukemia cytogenetic abnormalities, in particular t(8,21), has been associated with a higher incidence. Myeloid sarcoma, which rarely happens in acute promyelocytic leukemias, is more common in recurrent patients after the advent of all-trans retinoic acid (ATRA) and are rare in untreated acute promyelocytic leukemia. We described a case of, to our knowledge, *de novo* myeloid sarcoma of the spine confirmed as acute promyelocytic leukemia. Myeloid sarcoma is diagnosed by spinal tumor biopsy, and microscopic examination of a bone marrow smear and cytogenetic analysis led to a confirmed diagnosis of acute promyelocytic leukemia.

## Introduction

Myeloid sarcoma (MS), also known as granulocytic sarcoma or chloroma, is a rare extramedullary tumor of immature myeloid cells (1). MS can occur in patients with acute myeloid leukemia (AML), myelodysplastic syndrome, or chronic myelogenous leukemia. The incidence of MS in AML patients is 2%–10.4%, and most cases are associated with AML FAB M2, M4, and M5 (2, 3). Certain known acute myeloid leukemia (AML) cytogenetic abnormalities, in particular t(8,21), had been associated with a higher incidence of MS (4). The prevalence of myeloid sarcoma in any organ was 2.9% among all patients with acute and chronic myeloid leukemia. Furthermore, the prevalence of myeloid sarcoma of the spine was 1.0% among all patients with acute and chronic myeloid leukemia (5, 6). MS is more common in recurrent acute promyelocytic leukemia (APL) patients after the advent of all-trans retinoic acid (ATRA) but rare in untreated APL patients (7–9). Here, we describe a 50-year-old female patient that shows an MS involving the spine as the first manifestation of APL.

## Case Report

**Figure 1:**
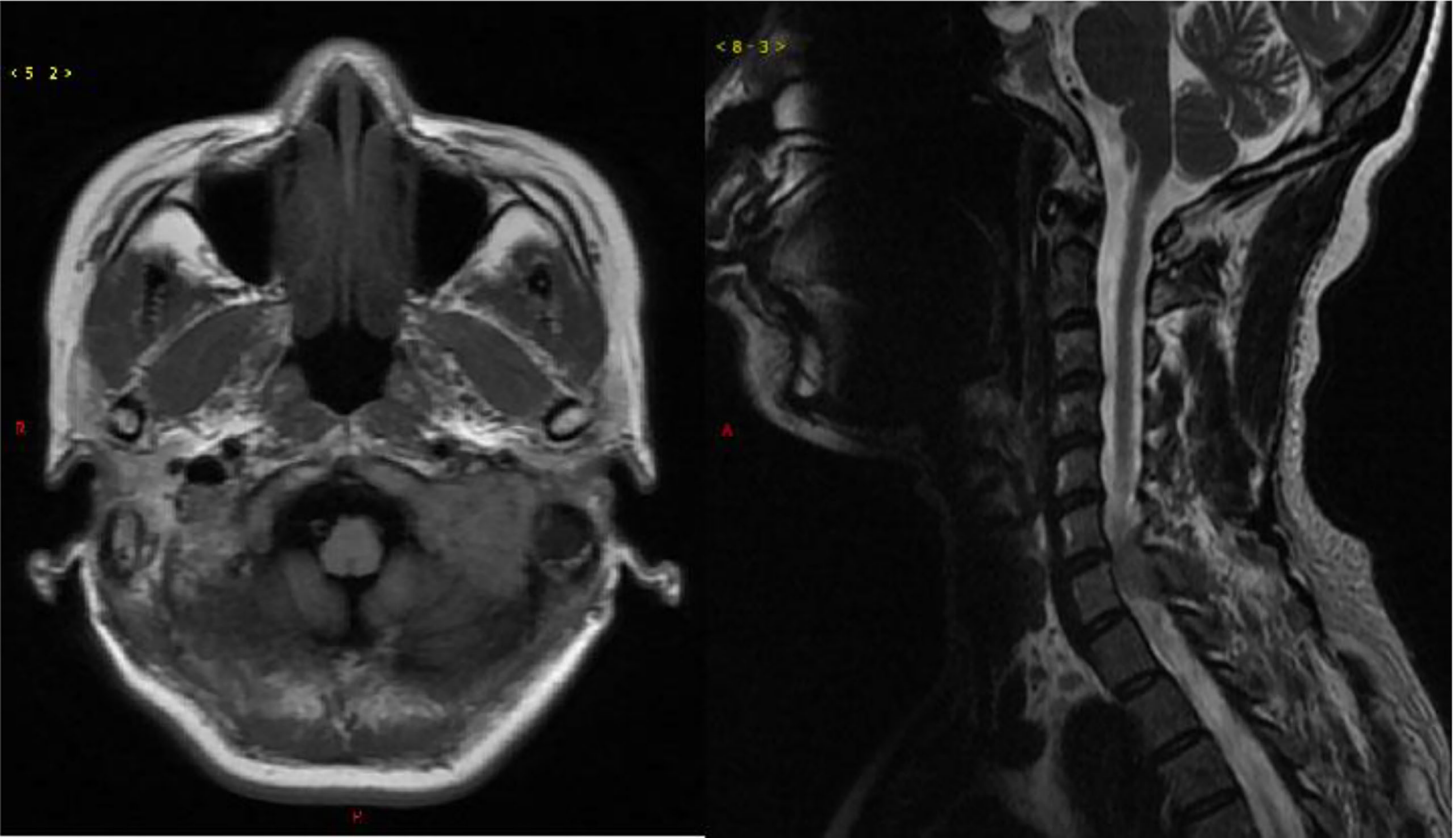
Magnetic resonance imaging of the brain and spine. Left, axial T1 weighted; right, sagittal T2 weighted.

**Figure 2:**
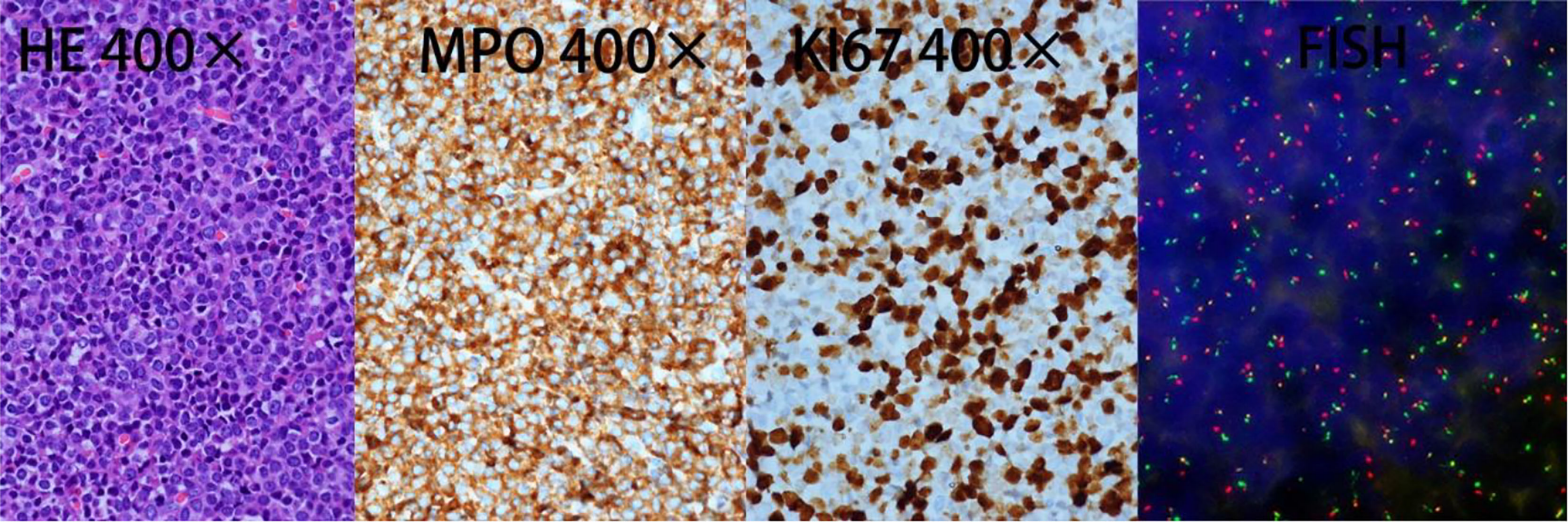
Histological analysis of the spine biopsy. HE, hematoxylin–eosin stain; MPO, myeloperoxidase positivity; KI67, proliferation index. FISH analysis performed on excised tumor demonstrated PML-RARα fusion.

In March 2021, a 50-year-old woman presented with paresthesia in her right hand and difficulty walking for more than a week. On examination, there was no any visible or palpable para-vertebral soft tissue mass in cervico -thoracic region and no paraparesis with a power of 5/5 in both lower limbs. Magnetic resonance imaging (MRI) showed a space occupation lesion (0.7 × 2.0 × 2.9 cm^3^) in the cervical vertebra canal from C6 through C7. Lower intensity on T1-weighted imaging, equal intensity on T2-weighted imaging, and obvious enhancement were observed in the lumpy lesion of the left occipital area (**Figure 1**). Then, intraspinal tumor resection and spinal Galveston internal fixation was conducted. During the operation, the mass was seen around C6–7 and grew outward toward the right intervertebral foramen of C6–T1. Histopathological examination of the mass revealed the accumulation of immature lymphohematopoietic cells. Immunohistochemical staining showed tumor cells with MPO (+), TDT (+), CD56 (+), CD43 (+), and Ki67 (Li: about 60%). *In situ* hybridization detection of EBV showed EBER (−) (**Figure 2**).

Routine examination results in hospitalization showed abnormal blood coagulation with a decrease in fibrinogen (1.23 g/L). The peripheral blood (PB) showed decreased parameters of white blood cells (WBCs) of 1.48 × 10^9^/L, neutrophils of 0.8 × 10^9^/L, and normal parameters of hemoglobin (Hb) of 120 g/L and platelets (PLT) of 159 × 10^9^/L. Liver and kidney functions and liver, spleen, and lymph node ultrasound showed no obvious abnormalities. Lung CT scan results revealed pulmonary infection and Insect-etch bone destruction in the posterior segment of the sixth costal axillary on the left.

**Figure 3:**
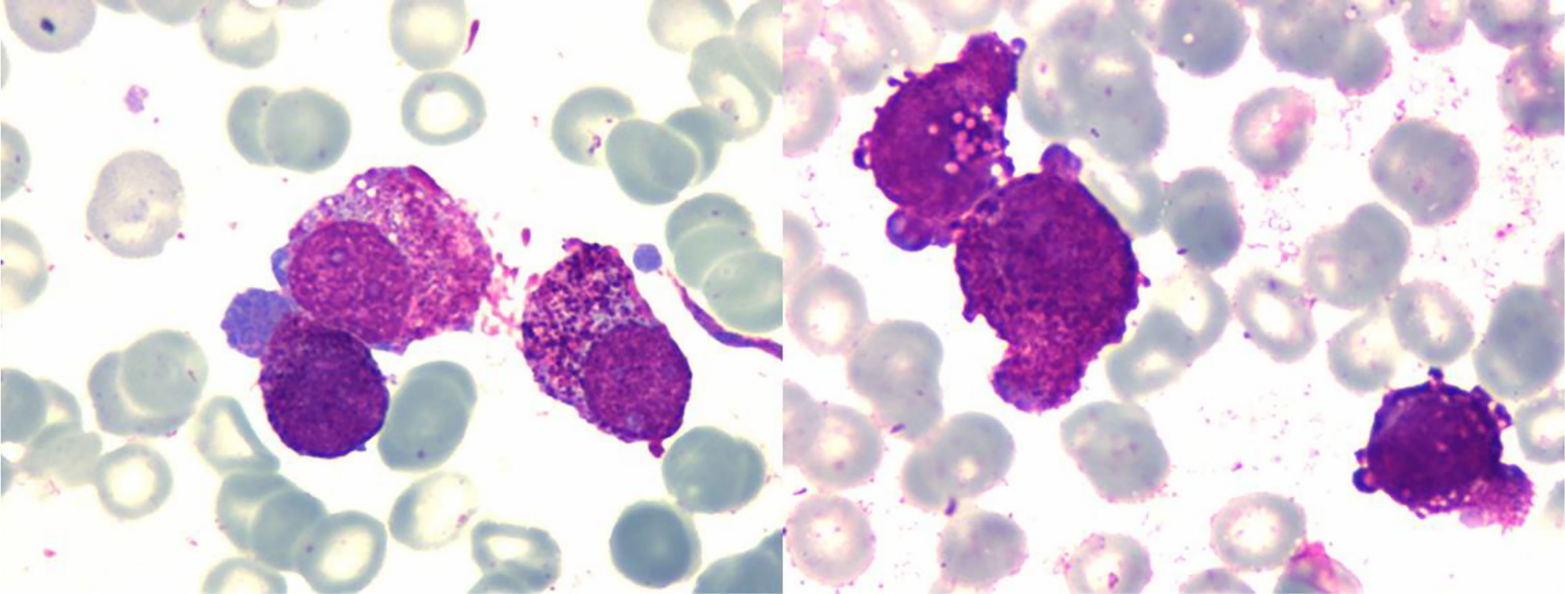
Promyelocytes contained round nuclei and abundant cytoplasm, but no Auer rods.

After 14 days of surgery, peripheral blood (PB) film examination showed 13% promyelocytes, and bone marrow (BM) aspirate showed that granulocytes accounted for 70%, with 50% promyelocytes that varied in diameter and contained rich cytoplasm with abundant small azurophilic granules. However, these leukemic cells did not contain Auer rods (**Figure 3**). The immunophenotype profile of leukemic cells was consistent with APL (CD117+, MPO+, CD33+, CD38 part+, CD13part+, CD64 part+, CD123 part+, CD15dim+, CD34−, CD19−, CD7−, CD3−, HLA-DR−, cCD79a−, CD16−, and CD56-). In addition, positive PML-RARα gene rearrangement in bone marrow was confirmed by RT-PCR. Single nucleotide variation (SNV) and small fragment insertion/deletion (InDel) were detected in RUNX1, FLT3, and KMT2C genes, and no FLT3-ITD mutation was detected through high-throughput sequencing (NGS). Whole-exome sequencing in the bone marrow and the nails were conducted, but no obvious abnormal gene mutations were found. Retrospective fluorescence *in situ* hybridization (FISH) testing for the PML-RARα fusion gene was conducted on samples of excised tumor in March and showed positive results with t(15:17)(q24;q21) as well (**Figure 2**).

After the diagnosis was confirmed in the hematology department, treatment began with induction regimen composed of ATRA 10 mg three times a day and Arsenic trioxide (ATO) 10 mg daily, from day 1 until complete remission, combined with idarubicin 10 mg on days 2, 4, and 6. Bone marrow evaluation was performed after the induction therapy and showed complete remission with a reduction of PML–RARα/ABL1 to 0.29%. Intrathecal chemotherapy with methotrexate and dexamethasone was performed four times after complete remission. After 2 courses of consolidation chemotherapy with idarubicin and cytarabine, repeat cranial and cervical MRI analysis showed the lumpy lesion of left occipital area was complete regression. Then the maintenance therapy had started with the drugs of ATRA and Compound Huangdai tablets. After 10 months of follow-up, the patient still remained in complete hematologic and molecular remission.

## Discussion

As a special type of AML, APL is characterized by a block in differentiation where leukemic cells are halted at the promyelocyte stage, accounting for roughly 5%–8% of AML patients. The characteristic balanced translocation t(15;17) (q22;q12) is seen in 95% of cases, which results in the fusion transcript PML-RARα. We summarized the literature of APL with MS as the initial presentation published so far; a total of 21 articles with 22 cases are included (10–30). It was found that this type of leukemia affects patients at a wide age range, from 1 to 77 years old. The average age of onset is 36 years, mostly in the young and middle aged. In terms of gender, there is no significant difference between men and women. The most common sites of APL with MS are spine (five cases), skin (three cases) and tongue (two cases), followed by the testis, axilla, breast, cerebellum, colon, femur, tibia, mandible, oral cavity, ovary, pelvis, rectum, and thymus. In terms of myeloid sarcoma of the spine, spinal cord compression is the initial presentation, which is similar to this patient. Besides, most APL with MS cases did not present with characteristic hemorrhage of APL. Therefore, a small number of cases are first misdiagnosed as a lymphoma or Ewing sarcoma due to insufficient immunophenotyping of the tumor tissue. Proper histological, flow cytometric, and cytogenetic analyses are essential for the correct diagnosis.

Most APL with MS cases are associated with PML-RARα fusion and chromosomal abnormalities in the bone marrow or MS tissue. The fusion transcript PML-RARα is detected in most cases (17/22). Only one case for NPM1-RARα fusion, one case for NPM-RARα, and two cases for FLT3-ITD mutation were found. Aside from the expression of the characteristic PML-RARα fusion protein, other co-occurring mutations including FLT3, WT1, NRAS, KRAS and ARID gene have also been noted in primary and relapse APL with MS. FLT3, has been shown to be the most commonly mutated gene in primary APL. Additionally, other commonly known AML mutations such as NPM1 are rarely seen in APL (31). It is speculated that FLT3-ITD plays a significant role in relapse and central CNS involvement in patients with APL (32). Several analyses found that FLT3-ITD mutation is associated with high WBC count at diagnosis and poor prognosis in patients with APL (33–36). NPM-RARα arrests myeloid differentiation at the promyelocyte stage like PML-RARα. APL carrying NPM-RARα shows a good response to differentiation therapy with all-trans retinoic acid (12, 22, 37). In this case, single nucleotide variation and small fragment insertion/deletion of RUNX1, FLT3, and KMT2C genes are involved. Besides FLT3 gene, RUNX1 is important in maintaining normal hematopoiesis and preventing the development of malignancy (38). KMT2C gene is a putative tumor suppressor in several epithelia and in myeloid cells (39). Therefore, the two gene mutations can also lead to the development of tumors. But since only single nucleotide variation and small fragment insertion/deletion rather than gene mutation are detected in this case, it is still unclear whether such changes play an important role in the pathogenesis.

According to an updated recommendations from an Expert Panel of the European Leukemia Net, ATRA-ATO combination induction and consolidation therapy have become the standard chemotherapy for low- to intermediate-risk patient (WBC ≤ 10 × 10^9^/L), which is associated with significantly less myelosuppression and fewer infections. Besides, in some countries, the classical combination of ATRA and chemotherapy is still an acceptable option, especially for high-risk patients with WBC>10 x 10_9_/L (40). Of the 22 cases of APL with MS, most cases (14/22) were non-high-risk patients (WBC≤ 10 x 10^9^/L) and the others (7/22) were high-risk patients. In high-risk patients, 3 patients received ATRA with chemotherapy, 2 received chemotherapy alone, 1 received ATRA with ATO and 1 received ATRA with ATO plus chemotherapy. Among them, 3 patients finally died from cerebral hemorrhage, CNS infiltration and hematological relapse, the others achieved complete or molecular remission. In non-high-risk patients, nine patients received ATRA with chemotherapy, four patients received ATRA with ATO plus chemotherapy, and one patient was given ATRA with ATO. Finally, two patients died from severe hemorrhagic episodes and sepsis, respectively, two cases eventually relapsed, and the others received complete molecular or hematological remission. It seemed that APL with MS were more frequently found in non-high-risk patients, and a poor prognosis was associated with high WBC counts. WBC count is lower than 10 × 10^9^/L in our patient who received ATRA with ATO plus chemotherapy induction therapy, and following three courses of chemotherapy, complete remission had been attained and the lumpy lesion of left occipital area disappeared.

Patients with APL frequently manifest as an coagulation abnormalities, which always present with decreased PLT counts and low serum fibrinogen levels (41). In most APL with MS case reports, serum fibrinogen levels were not described. In patients of APL with MS, except for 1 case without description of PLT counts, 10 of 22 patients showed low PLT counts, 11 of 22 patients showed normal PLT counts. No significant hemorrhagic episodes were observed in all patients with normal PLT counts. In patients with low PLT counts, minor bleeding such as gingival, nose, and rectal bleeding were seen in four patients. Life threatening severe bleeding was seen in two patients. From these case reports, the risk of bleeding in APL presenting with MS patients were not high, and the prognosis is good, in general. But in five death cases, two of them died of bleeding; bleeding complications are still the common leading cause of death in these cases. In our patient, PLT counts were normal and fibrinogen declined lightly, and no obvious bleeding was observed during the whole treatment.

Five cases of APL presenting with a myeloid sarcoma in the spine were all non-high-risk patient, mostly occurring in male (4/5). The thoracic spine was involved in all cases, followed by the lumbar, which was consistent with our case. One responder died of sepsis while still in hematological remission, and the remaining four achieved consistent remission. Four of five patients were treated with radiotherapy; no significant radiotherapy effects were found. Radiotherapy alone seemed to be ineffective in some cases (18, 19, 21, 22, 24). One retrospective study demonstrated that majority of patients that presented with MS had not received radiotherapy because the mass regressed after induction chemotherapy. The majority of patients with MS were referred for radiotherapy when there was extramedullary progression, marrow relapse, or rapid symptom relief required (42). However, there are no specific studies on the efficacy of radiotherapy for myeloid sarcomas of the spine (42, 43).

In conclusion, the clinical manifestation of APL with MS is different from characteristic APL and first identified by tumor biopsy but easily misdiagnosed because of atypical morphology. Further microscopic examination of bone marrow smear and cytogenetic analysis plays an important role to determine the source of MS, which are also very vital for subsequent treatment.

## Data Availability Statement

The original contributions presented in the study are included in the article/supplementary material. Further inquiries can be directed to the corresponding author.

## Author Contributions

XS, QW, and JL took care of the patient. XS and QW drafted the manuscript. XS, TG, and HY collected clinical data. QW and JL analyzed the review data and critically revised the manuscript. All authors contributed to the article and approved the submitted version.

## Funding

This study was supported by the National Natural Science Foundation of PR China (no. 81770219, for QW).

## Conflict of Interest

The authors declare that the research was conducted in the absence of any commercial or financial relationships that could be construed as a potential conflict of interest.

## Publisher’s Note

All claims expressed in this article are solely those of the authors and do not necessarily represent those of their affiliated organizations, or those of the publisher, the editors and the reviewers. Any product that may be evaluated in this article, or claim that may be made by its manufacturer, is not guaranteed or endorsed by the publisher.
